# Using satellite data to assess spatial drivers of bird diversity

**DOI:** 10.1002/rse2.322

**Published:** 2022-12-24

**Authors:** Merryn L. Hunt, George Alan Blackburn, Gavin M. Siriwardena, Luis Carrasco, Clare S. Rowland

**Affiliations:** ^1^ UK Centre for Ecology & Hydrology, Lancaster Environment Centre Lancaster University Lancaster LA1 4YQ United Kingdom; ^2^ Lancaster Environment Centre Lancaster University Lancaster LA1 4YQ UK; ^3^ British Trust for Ornithology, The Nunnery, Thetford Norfolk IP24 2PU United Kingdom; ^4^ Descartes Labs, Inc. Santa Fe New Mexico USA

**Keywords:** avian, feature contribution analysis, habitat productivity, Landsat, landscape heterogeneity, random forest

## Abstract

Birds are useful indicators of overall biodiversity, which continues to decline globally, despite targets to reduce its loss. The aim of this paper is to understand the importance of different spatial drivers for modelling bird distributions. Specifically, it assesses the importance of satellite‐derived measures of habitat productivity, heterogeneity and landscape structure for modelling bird diversity across Great Britain. Random forest (RF) regression is used to assess the extent to which a combination of satellite‐derived covariates explain woodland and farmland bird diversity and richness. Feature contribution analysis is then applied to assess the relationships between the response variable and the covariates in the final RF models. We show that much of the variation in farmland and woodland bird distributions is explained (*R* ^2^ 0.64–0.77) using monthly habitat‐specific productivity values and landscape structure (FRAGSTATS) metrics. The analysis highlights important spatial drivers of bird species richness and diversity, including high productivity grassland during spring for farmland birds and woodland patch edge length for woodland birds. The feature contribution provides insight into the form of the relationship between the spatial drivers and bird richness and diversity, including when a particular spatial driver affects bird richness positively or negatively. For example, for woodland bird diversity, the May 80th percentile Normalized Difference Vegetation Index (NDVI) for broadleaved woodland has a strong positive effect on bird richness when NDVI is >0.7 and a strong negative effect below. If relationships such as these are stable over time, they offer a useful analytical tool for understanding and comparing the influence of different spatial drivers.

## Graphical abstract

The aim of this paper is to understand the importance of different spatial drivers for modelling bird distributions. Specifically, it assesses the importance of satellite‐derived measures of habitat productivity and landscape heterogeneity for modelling bird diversity across Great Britain. Random Forest (RF) regression is used to explore the extent to which a combination of satellite‐derived covariates explain woodland and farmland bird diversity, and feature contribution analysis is then applied to explore the relationships between the response variable and the covariates in the final RF models.

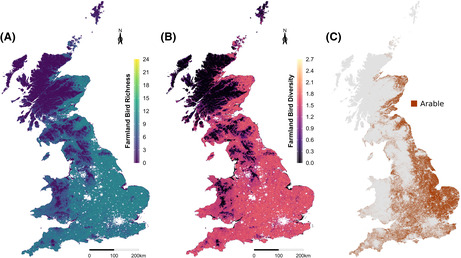

## Introduction

Despite targets to reduce its loss, global biodiversity has continued to decline as pressures have increased (Butchart et al., 2010; Díaz et al., 2019; IBPES, 2019). Biodiversity loss has been linked to large changes in primary productivity, decomposition, and other ecosystem services (Hooper et al., 2012). Understanding the spatial drivers of biodiversity patterns is essential to predict how different species may respond to future environmental changes and inform the creation of effective conservation strategies. To achieve this, comprehensive information is required regarding species distribution and changes over time, and the spatial drivers of these changes.

There is a large body of research dedicated to demonstrating the potential of using remote sensing, including satellite data, to monitor biodiversity (e.g. Gottschalk et al., 2005; Kuenzer et al., 2014; Pettorelli et al., 2014; Rocchini et al., 2016). Satellite images provide a readily accessible, global dataset at various spatial and temporal resolutions from which indicators of bird species diversity—an important indicator of global biodiversity patterns (Furness & Greenwood, 2013)—may be derived (e.g. Kerr & Ostrovsky, 2003; Nagendra, 2001; Tuanmu & Jetz, 2015; Turner et al., 2003). Previous studies have mapped bird diversity using satellite‐derived measures of two key factors affecting species diversity: (1) spatial heterogeneity, including measures of habitat structure, composition and connectivity (Carrasco et al., 2018; Coops, Wulder, et al., 2009; Griffiths & Lee, 2000; Luoto et al., 2004), and (2) environmental productivity, measured using fraction of absorbed photosynthetically active radiation (fAPAR) (Coops, Waring, et al., 2009), gross and net primary productivity (GPP/NPP) (Phillips et al., 2008, 2010) and normalized difference vegetation index (NDVI) (Duro et al., 2014; Foody, 2005; Seto et al., 2004). Research has particularly focused on assessing the impact of different covariates on the accuracy of bird richness mapping, including assessing the value of image texture (Hepinstall & Sader, 1997; St‐Louis et al., 2006; Tuanmu & Jetz, 2015), the sensitivity to timing of MODIS images (Bonthoux et al., 2018), the inclusion of climate data alongside the satellite‐based covariates (Carroll et al., 2022; Thuiller et al., 2004) and developing new indices (Coops, Waring, et al., 2009).

In terms of scale, work to date has ranged from global and national‐scale with MODIS (e.g. Coops, Wulder, et al., 2009; Hobi et al., 2017; Tuanmu & Jetz, 2015), regions within countries using Landsat (St‐Louis et al., 2014) and recently national‐scale mapping with Landsat (Carroll et al., 2022; Farwell et al., 2020). Use of higher resolution data, such as Landsat and Sentinel‐2, allows EO‐based covariates to be derived at scales more suitable for detecting smaller‐scale, within‐habitat variations, thus potentially improving the accuracy with which bird diversity can be mapped; this is particularly true for countries, or areas within countries, that have mixed land‐cover at the scale of a MODIS pixel (Hill & Smith, 2005). Historically, this has been hindered by the computing resources required for national scale analysis. Now, with recent advances in machine learning and the advent of cloud computing platforms such as Google Earth Engine (Gorelick et al., 2017), this analysis is possible (Carroll et al., 2022; Farwell et al., 2020). Recent years have also seen increased quality and consistency of satellite datasets for automated analysis, with improved georeferencing and cloud‐masking (Roy et al., 2014). These developments make it timely to assess the spatial drivers of bird richness and diversity at higher resolution.

Random forest regression provides a flexible and robust method for ecological analysis, dealing with missing values and complex nonlinear relationships among predictor variables (Cutler et al., 2007). Random forest regression (RFR) has been used to assess the response of bird species richness to environmental heterogeneity (Carrasco et al., 2018), to predict rare and invasive species presences (e.g. Cutler et al., 2007; Lawrence et al., 2006; Mi et al., 2017; Prasad et al., 2006) and in land cover mapping (e.g. Gislason et al., 2006; Rodriguez‐Galiano et al., 2012). However, as a black box modelling approach, additional analysis such as feature contribution is required to assess the relationships between predictor and response variables (Kuz'min et al., 2011; Palczewska et al., 2014), and thus assess the drivers of spatial and temporal variations in species. Understanding these drivers in different locations and at different scales has the potential to provide valuable insight into critical factors affecting species diversity, abundance and richness.

In this paper, we model bird diversity across Great Britain (GB) using Landsat data and determine the drivers of observed spatial variation. This builds on previous work (Bonthoux et al., 2018; Carrasco et al., 2018; Fuller et al., 2005, 2007) and complements recent studies (Carroll et al., 2022) by incorporating Landsat‐derived measures of habitat extent, and monthly heterogeneity and productivity into the modelling. To do this, RFR trained using bird survey data was used to assess the extent to which a combination of satellite‐derived measures of habitat heterogeneity and habitat productivity could explain the variation of bird diversity across GB. Variable selection techniques were used to reduce the number of variables and produce a set of refined RFR models. These models were then used to produce national scale predictive maps of farmland and woodland bird diversity and richness. Finally, feature contribution analysis was used to demonstrate how the nature of relationships between the response and predictor variables in the refined RFR models can be assessed.

## Materials and Methods

**Figure 1:**
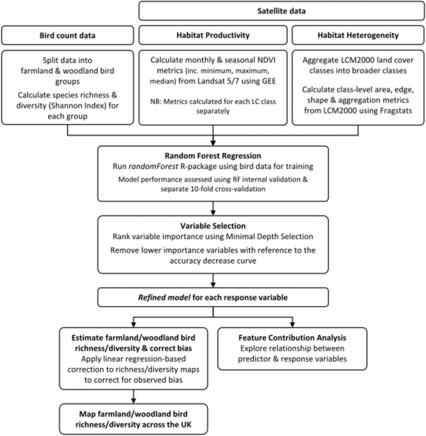
Overview of the method used to estimate bird diversity distributions across GB.

Figure 1 provides an overview of the method used in this study, outlining how the bird count data and satellite data were processed and combined to estimate bird diversity distributions across GB. Firstly, farmland and woodland bird species richness and diversity were derived from bird count data. Then measures of habitat heterogeneity were derived from United Kingdom (UK) Land Cover Map 2000, while Landsat data were used to derive monthly and seasonal NDVI metrics, as a proxy for habitat productivity. These data were used to train RFR models, refined using variable selection techniques, which were used to predict farmland and woodland bird species richness and diversity and assess spatial drivers using feature contribution analysis.

### Bird data

Bird count data were collected between April and June of 2000 in 335 UK Countryside Survey 2000 (CS2000) squares (Wilson & Fuller, 2002) distributed across GB. The UK Countryside Survey squares are a stratified, random sample of 1 km squares covering GB based on an environmental stratification to ensure a representative sample (Firbank et al., 2003). Bird counts were recorded on two separate visits during the early and late breeding season, using up to 4 km of line transect counts per square. The surveying methodology followed that of the British Trust for Ornithology (BTO)/Joint Nature Conservation Committee (JNCC)/Royal Society for the Protection of Birds (RSPB) Breeding Bird Survey (Harris et al., 2019). In our study, we used species richness and the Shannon diversity index as measures of farmland and woodland bird species diversity, based on the maximum counts per species across visits. The designation of farmland and woodland species followed the groupings developed for the BTO/JNCC/RSPB UK Wild Bird Population indicators (Eaton & Noble, 2018). The indicator for breeding farmland bird populations includes grassland and grazing pastures in the definition of farmland. Farmland and woodland bird richness were calculated for each square by counting the number of species along the transects.

### Habitat heterogeneity variables

There are many measures of environmental heterogeneity (Stein et al., 2014), however, here the focus is on land cover heterogeneity, defined as between‐habitat heterogeneity (Stein et al., 2014), which can be readily calculated from land cover data. Measures of habitat heterogeneity, including habitat extent, patch area and edge length, were derived from the UK Land Cover Map 2000 (LCM2000; Fuller, Smith, Sanderson, et al., 2002) within each 1 km square using FRAGSTATS v4 (McGarigal et al., 2012). LCM2000 was derived from image segmentation of Landsat data and has a minimum mappable unit of 0.5 ha; as a parcel‐based land cover product, it is well suited for calculating habitat heterogeneity values (Fuller, Smith, Sanderson, et al., 2002). To calculate the landscape variables, the land cover classes were converted from the original 26 classes into a smaller set of aggregated land cover classes (Table S1 in Appendix S1). FRAGSTATS metrics were calculated for the arable, broadleaved, coniferous, grassland and semi‐natural aggregated land cover classes (see Table S2 in Appendix S1 for a full list of metrics). Descriptions (adapted from Mcgarigal (2015)) of the key metrics for the final models can be found in Table 1.

**Table 1:** Descriptions of the key FRAGSTATS metrics for the final richness and diversity random forest model (adapted from McGarigal, 2015).

FRAGSTATS metrics	Description of metric
Percentage of landscape (PLAND)	The percentage of each square comprised of a particular class.
Effective mesh size (MESH)	Quantifies habitat fragmentation based on the probability that two randomly chosen points in the region under interest are located in the same non‐fragmented patch. The probability is multiplied by the total area of the landscape unit. The more barriers (e.g. roads, railroads) in the landscape, the lower the probability that the two locations will be located in the same patch, and the lower the effective mesh size.
Contiguity Index (CONTIG)	Measure of spatial connectedness/contiguity of cells within a grid‐cell given as the mean (MN), coefficient of variation (CV) or area‐weighted mean (AM) per class. An index value of zero represents a one‐pixel patch, increasing to 1 as connectedness increases.
Related circumscribing circle (CIRCLE)	Measure of overall patch elongation using the ratio of patch area to the ratio of the smallest circumscribing circle given as mean (MN), coefficient of variation (CV) or area‐weighted mean (AM) per class. Highly convoluted, but narrow patches give a low index value, while narrow and elongated patches have a high index value.
Patch cohesion (COHESION)	Provides a measure of the physical connectedness of the corresponding class. COHESION approaches zero as the proportion of the focal class decreases and becomes increasingly subdivided, and therefore less physically connected.
Largest Patch Index (LPI)	Quantifies the percentage of the total landscape area comprised by the largest patch of a class. The LPI approaches zero when the largest patch of the corresponding type is increasingly small. An LPI value of 100 indicates the entire landscape is one patch.
Total edge (TE)	Absolute measure of total edge length of a particular class.
Edge density (ED)	Edge length of a particular class standardized to a per unit area basis (m/ha).

### Habitat productivity variables

The NDVI was used as a proxy for habitat productivity, because it shows a strong positive correlation with net primary productivity (NPP) at latitudes and habitat types similar to those in GB (e.g. Boelman et al., 2003; Evans et al., 2005; Kerr & Ostrovsky, 2003; Tebbs et al., 2017). NDVI‐based habitat productivity metrics were calculated in Google Earth Engine (Gorelick et al., 2017) using data from Landsat‐5 and Landsat‐7. Greenest pixel composites were produced for each month from March to September for Landsat images from 1999 and 2000; these were used to produce monthly NDVI images. The Landsat‐5 and Landsat‐7 data were then merged by taking the maximum NDVI value for each pixel from the monthly NDVIs; creating monthly maximum value composites (MVCs). Atmospherically corrected Landsat‐5 and Landsat‐7 images have been shown to produce similar NDVI measurements (Thieme et al., 2020; Vogelmann et al., 2001); hence, no cross‐calibration was required before merging the datasets. Combining Landsat‐5 and Landsat‐7, along with linear interpolation of NDVI values, to fill cloud‐gaps in individual months, enabled relatively cloud‐free coverage of GB to be produced.

From the monthly MVCs, habitat‐specific NDVI metrics were calculated for each land cover class within each square, for each month and for the growing season (March–September) as a whole; areas of the different land cover classes were identified using LCM2000. The metrics calculated were mean, standard deviation, coefficient of variation, median, minimum, maximum, range, 20th percentile, 80th percentile, interquartile range and sum (growing season only) of the NDVI values. These metrics were only calculated for the arable, broadleaved, coniferous, grassland and semi‐natural aggregated land cover classes (Table S1).

### RFR models and variable selection

RFR (Breiman, 2001) was used to determine which habitat productivity and heterogeneity variables provide the highest estimation accuracy for farmland and woodland bird species richness and diversity. Three sets of models were created: *full models*, using all available variables; *important variable* models, containing variables identified by minimal depth selection; and *refined models*, featuring the smallest sub‐set of variables, produced after implementing feature selection. The *randomForest* package (Liaw & Wiener, 2002) in *R* was used to build and analyse the RFR models with 1000 trees. RFR was chosen because of its ability to handle non‐linear responses and complex interactions between variables (Breiman, 2001; Prasad et al., 2006).

Minimal depth selection (Ishwaran et al., 2010) was used to rank the explanatory power of each estimator variable within the RFR models, using the *randomForestSRC* package in R (Ishwaran & Kogalur, 2019). Minimal depth assumes that variables that split nearest to the root node have a higher impact on the estimation than variables that split nodes further down the tree. While it is possible for non‐estimative variables to split close to the root node and not impact estimation, such occurrences are rare in a large forest of trees and averaging minimizes their effects (Ishwaran et al., 2010). Variables not deemed important by minimal depth selection were excluded from the models.

**Figure 2:**
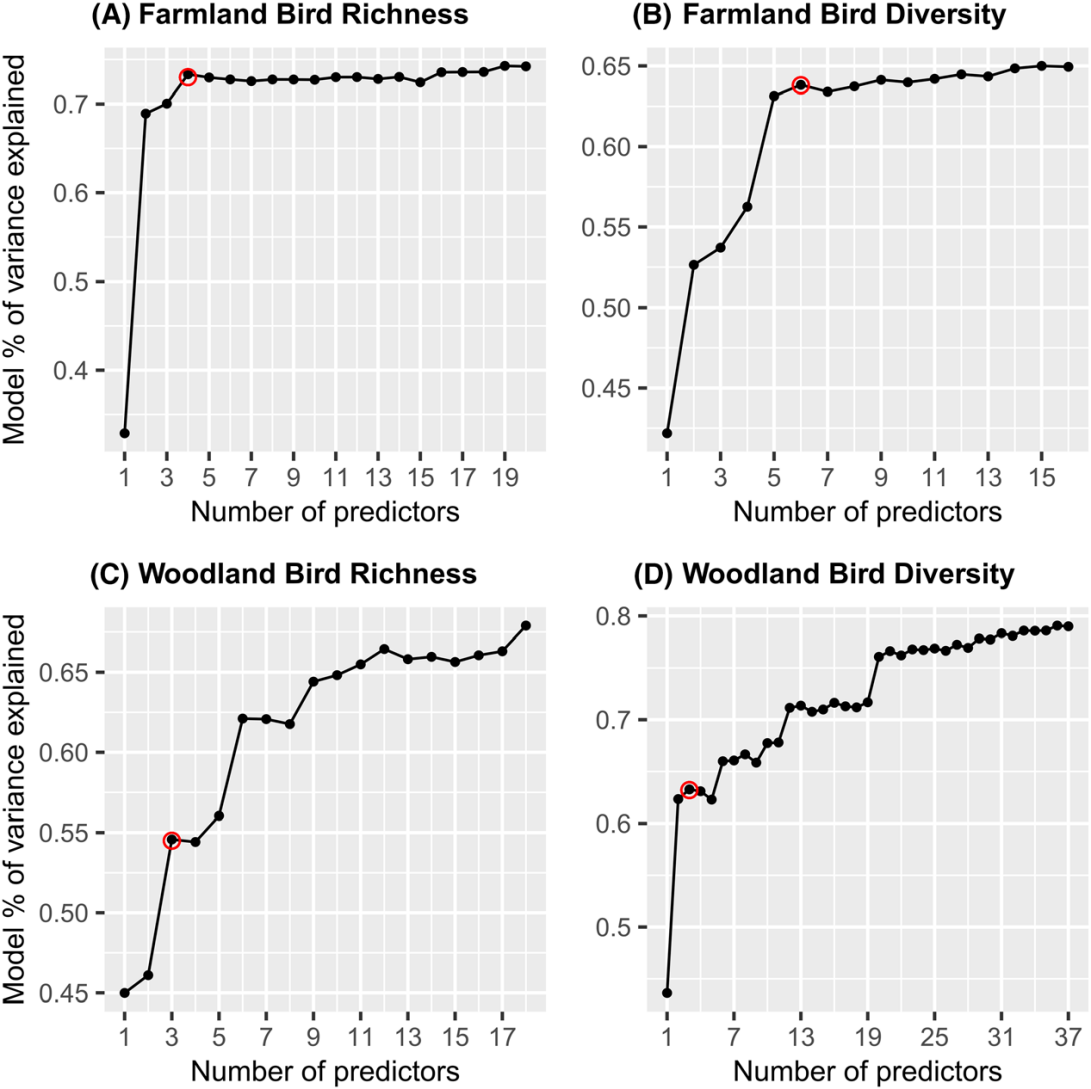
Variance explained (%) for the models including different number of predictors (by ranking), for (A) farmland bird richness, (B) farmland bird diversity, (C) woodland bird richness and (D) woodland bird diversity. The red dot indicates the point immediately before a decrease in accuracy is first observed as another predictor is added, indicating the number of predictors to be included in each refined model.

Feature selection was then used to reduce model complexity further and simplify interpretation of the final models. To identify the number of variables to include in each model, lower importance variables, as determined by the minimal depth selection, were excluded progressively and the change in variance explained by the model assessed. After removing all of the variables one by one, this produced accuracy curves (Fig. 2; Ishwaran et al., 2010). The number of variables included in each of the final refined RFR models was determined based on the point at which a decrease in accuracy (variance explained) was first observed in the accuracy curves following the addition of another estimator variable to the model.

The refined models were used to estimate farmland and woodland bird species richness and diversity across GB at 1 km resolution. All models were trained using data from the 335 CS2000 squares with bird count data. In the training data, the maximum percentage of urban cover was 54.6%; therefore, the final models were not applied to squares exceeding this level. A tendency was observed for RFR to underestimate the maximum value and overestimate the minimum value for each of the response variables. This was resolved by applying a linear regression‐based correction, to the maps produced by RFR, to adjust for the bias (Zhang & Lu, 2012).

The performance of the models built at each different stage of the variable selection process were compared using *R* ^2^ values calculated in two different ways: (i) internal validation carried out by the randomForest package, and (ii) a separate 10‐fold cross‐validation using the full training dataset.

### Feature contribution

To assess the relationship between the estimator and response variables in each of the final models, we used feature contribution analysis (Palczewska et al., 2014) using the *forestFloor* package in *R* (Welling et al., 2016). Feature contribution analysis reveals the magnitude and direction of influence (positive or negative) that estimator variables have on the response. This enables the feature contribution to be plotted for each estimator variable to understand the circumstances and extent to which it affects the response variable (Palczewska et al., 2014). Using *forestFloor*, we produced a series of plots, where the *y*‐axis represents the change of estimated bird richness for a given variable value, and the *x*‐axis represents the studied variable. A positive value on the *y*‐axis indicates a positive effect on richness or diversity, while a negative value indicates a negative effect; zero indicates no contribution.

## Results

### Model accuracies and predictive maps

The initial RFR models using all variables had 10‐fold cross validation *R* ^2^ values between 0.64 and 0.77 for farmland and woodland bird richness and diversity; while models containing only the important variables (identified using minimal depth selection) produced *R* ^2^ values between 0.65 and 0.80 (Table 2). A full list of the variables selected by minimal depth can be found in Table S3 in the Appendix S1, with Table S1 showing the level of correlation between variables.

**Table 2:** *R*‐squared values for (i) full RFR models containing all variables, (ii) important RFR models containing variables categorized as important by the minimal depth selection, and (iii) the final refined RFR models after applying feature selection to further reduce variable number. R‐squared values are given for the internal RFR validation and a separate 10‐fold cross validation.

Response variable	*R* ^2^ values					
	Full RFR model (all variables)		Model containing only important variables (# of variables)		Refined RFR model (# of variables)	
	Internal RFR validation	10‐fold cross validation	Internal RFR validation	10‐fold cross validation	Internal RFR validation	10‐fold cross validation
Farmland bird richness	0.73	0.77	0.75 (20)	0.73	0.73 (4)	0.78
Farmland bird diversity	0.65	0.64	0.65 (16)	0.69	0.64 (6)	0.59
Woodland bird richness	0.70	0.72	0.68 (18)	0.65	0.55 (3)	0.52
Woodland bird diversity	0.76	0.77	0.79 (37)	0.80	0.63 (3)	0.63

These models were then refined with feature selection to produce the final refined models used to understand the spatial drivers of bird diversity and richness and produce the final maps. This analysis revealed that the top 4 most important variables, according to minimal depth selection, were required for estimating farmland bird richness, the top 6 for farmland bird diversity, and top 3 for both woodland bird richness and diversity. Table 3 shows the variables included in each refined model. Subsequent analyses are based on these refined RFR models. The refined models had 10‐fold cross validation *R* ^2^ values between 0.52 and 0.78 (Table 2). The refined models all contain a mix of FRAGSTATS heterogeneity metrics, highlighting the importance of landscape configuration and extent, and habitat‐specific monthly vegetation productivity metrics.

**Table 3:** Variables used for the final refined RFR models after applying feature selection to further reduce variable number (see also Table 2).

Response variable	Variables (from most to least important)	Variables in final model
Farmland bird richness	March maximum NDVI for grassland Effective arable mesh size April 80th percentile NDVI for grassland July NDVI range for arable	FRAGSTATS: 1 NDVI: 3
Farmland bird diversity	April 80th percentile NDVI for grassland March maximum NDVI for grassland April maximum NDVI for grassland June maximum NDVI for grassland Arable contiguity index Arable related circumscribing circle	FRAGSTATS: 2 NDVI: 4
Woodland bird richness	Total broadleaved woodland edge length Broadleaved woodland edge density June maximum NDVI for broadleaved woodland	FRAGSTATS: 2 NDVI: 1
Woodland bird diversity	May 80th percentile NDVI for broadleaved woodland Semi‐natural largest patch index Broadleaved woodland percentage cover	FRAGSTATS: 2 NDVI: 1

**Figure 3:**
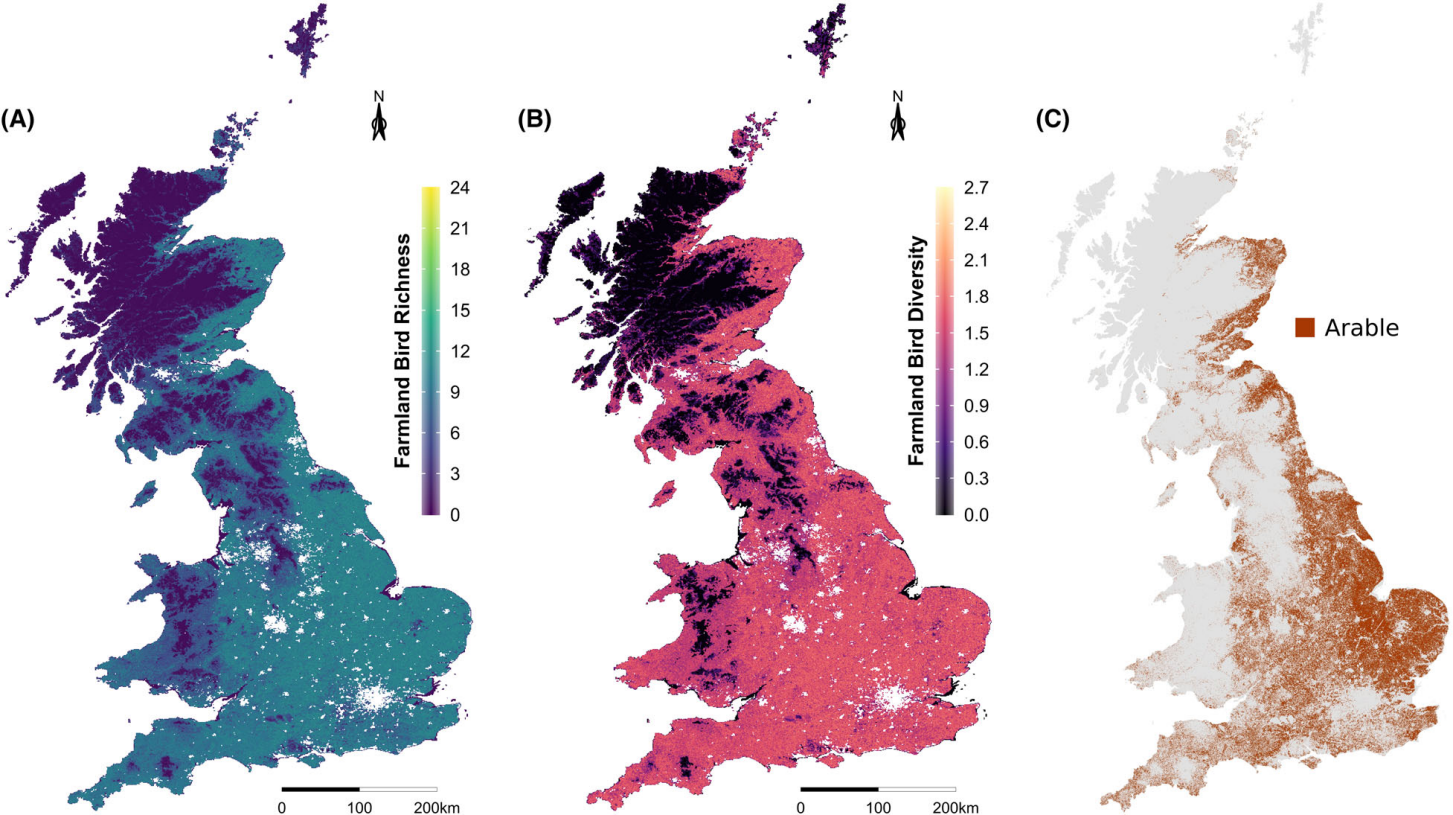
Predicted farmland bird (A) richness and (B) diversity (Shannon Index) maps at 1 km resolution, and (C) the distribution of arable land across GB according to LCM2000.

**Figure 4:**
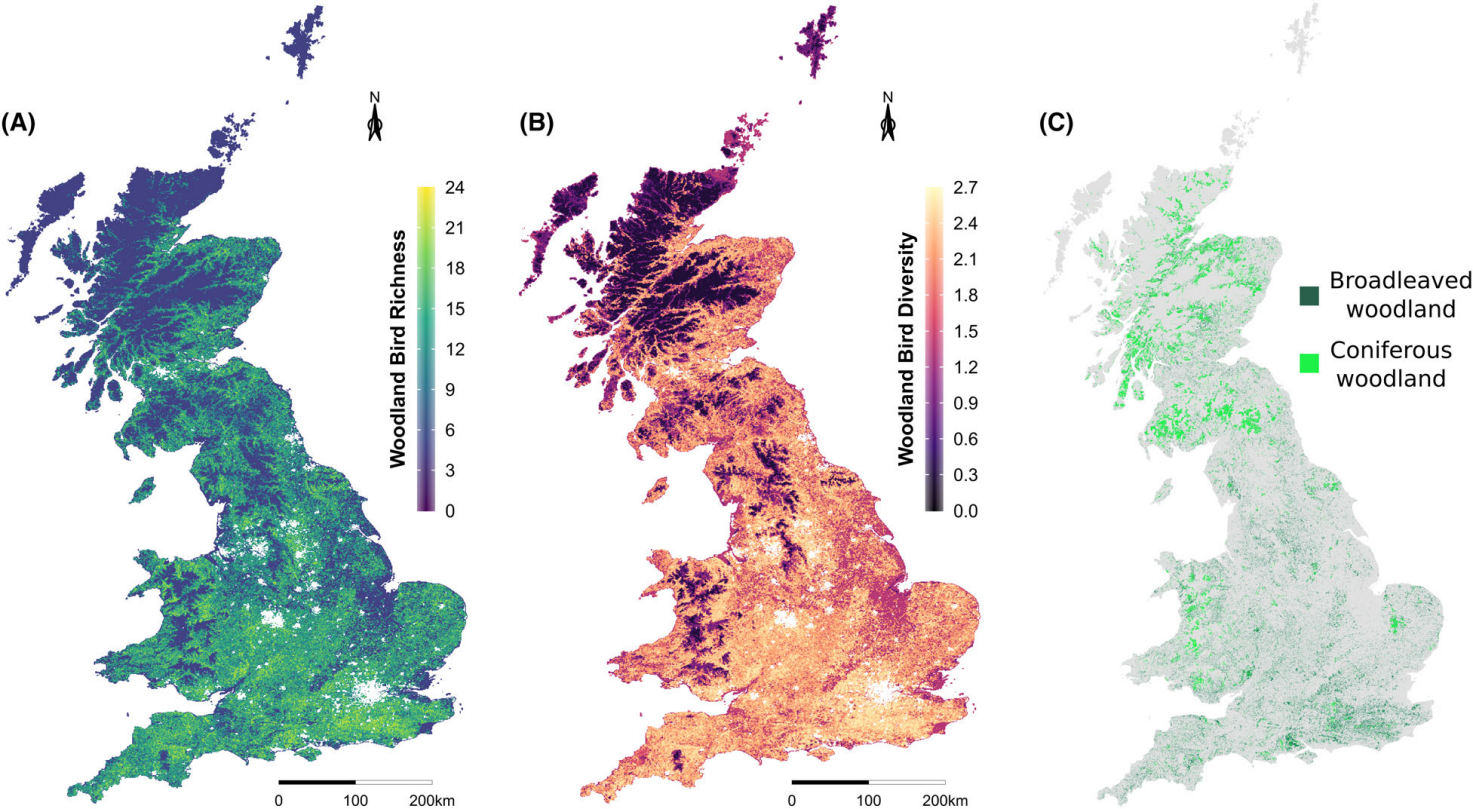
Predicted woodland bird (A) richness and (B) diversity (Shannon Index) maps at 1 km resolution, and (C) the distribution of broadleaved and coniferous woodland across GB according to LCM2000.

The refined RFR models were used to produce maps of farmland (Fig. 3) and woodland (Fig. 4) bird richness and diversity in 2000 for GB at a 1 km resolution. Figure 3A and B shows that farmland bird richness and diversity is highest in the east of England and lower in the west of England and in Scotland, a pattern which broadly matches the distribution of arable land across the UK. Figure 4A and B shows that levels of woodland bird richness and diversity appear to be more widely distributed around the UK, although there appears to be a prevalence for higher species richness and diversity in lowland areas and lower richness and diversity in highland areas.

Although a bias correction was applied, this did not completely account for the under‐estimation of maximum values and over‐estimation of minimum values. For example, the minimum value of woodland bird richness in the observed data was 0, while the lowest predicted value was 3. The fact that the predicted values do not currently fully reflect what we see in the bird count data may suggest that the current predictor variables do not capture all the important environmental factors affecting bird species richness and diversity. For example, linear landscape features, such as hedgerow habitats, are known to be important (Aue et al., 2014; Hinsley & Bellamy, 2000; Morelli et al., 2014; Sullivan et al., 2017), but were not included here.

### Feature contribution (for the refined models)

**Figure 5:**
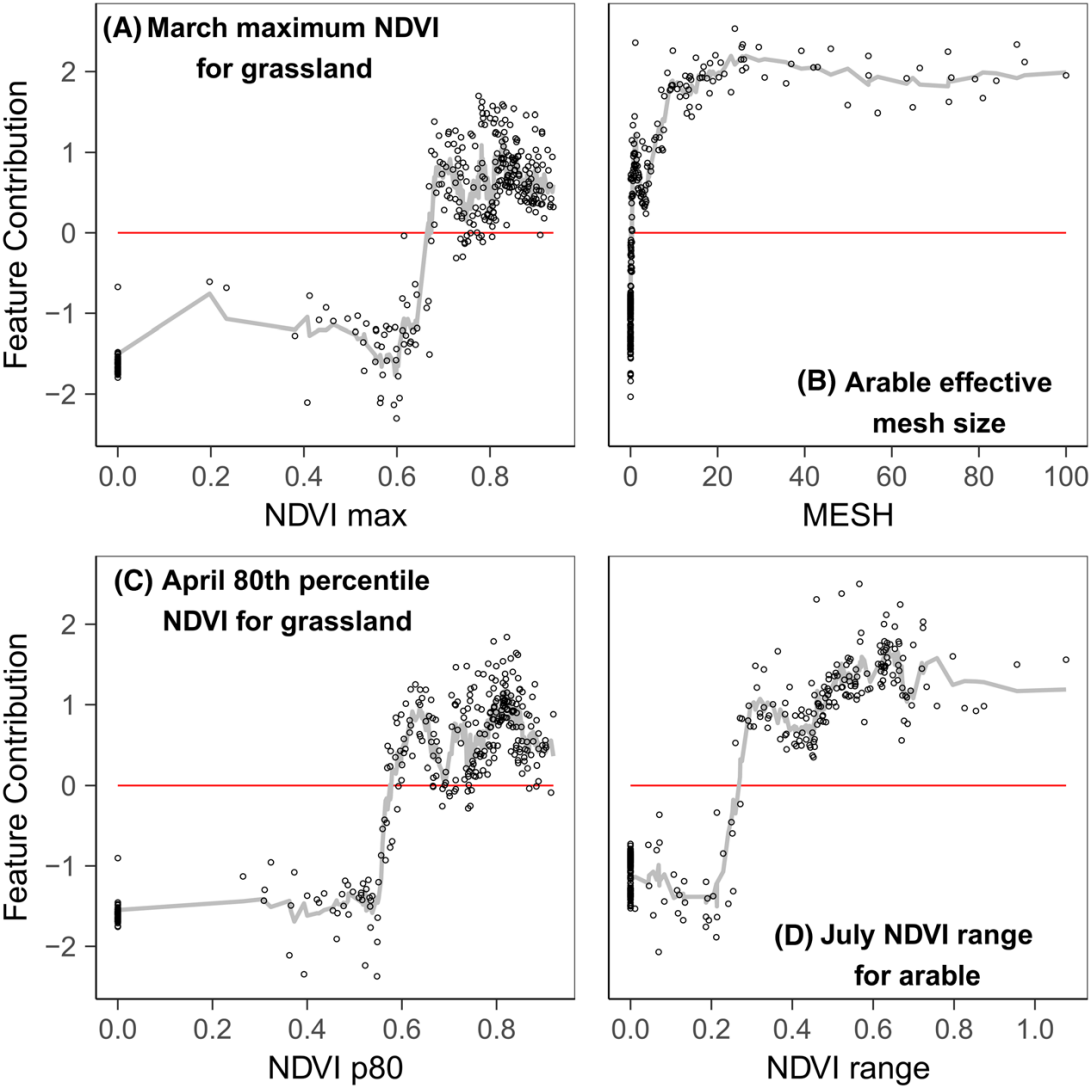
FC plots for the farmland bird richness prediction model variables: (A) March maximum NDVI for grassland, (B) arable effective mesh size, (C) April 80th percentile NDVI for grassland, and (D) July NDVI range for arable. The *y*‐axis represents the change of predicted bird richness for a given variable value, measured with the cross‐validated FC. The *x*‐axis represents the studied variable. The fitted line is based on the *k*‐nearest neighbour (knn) estimations. The red line indicates the point of zero FC. Zero values on the *x*‐axis denote the absence of the metric from the square.

**Figure 6:**
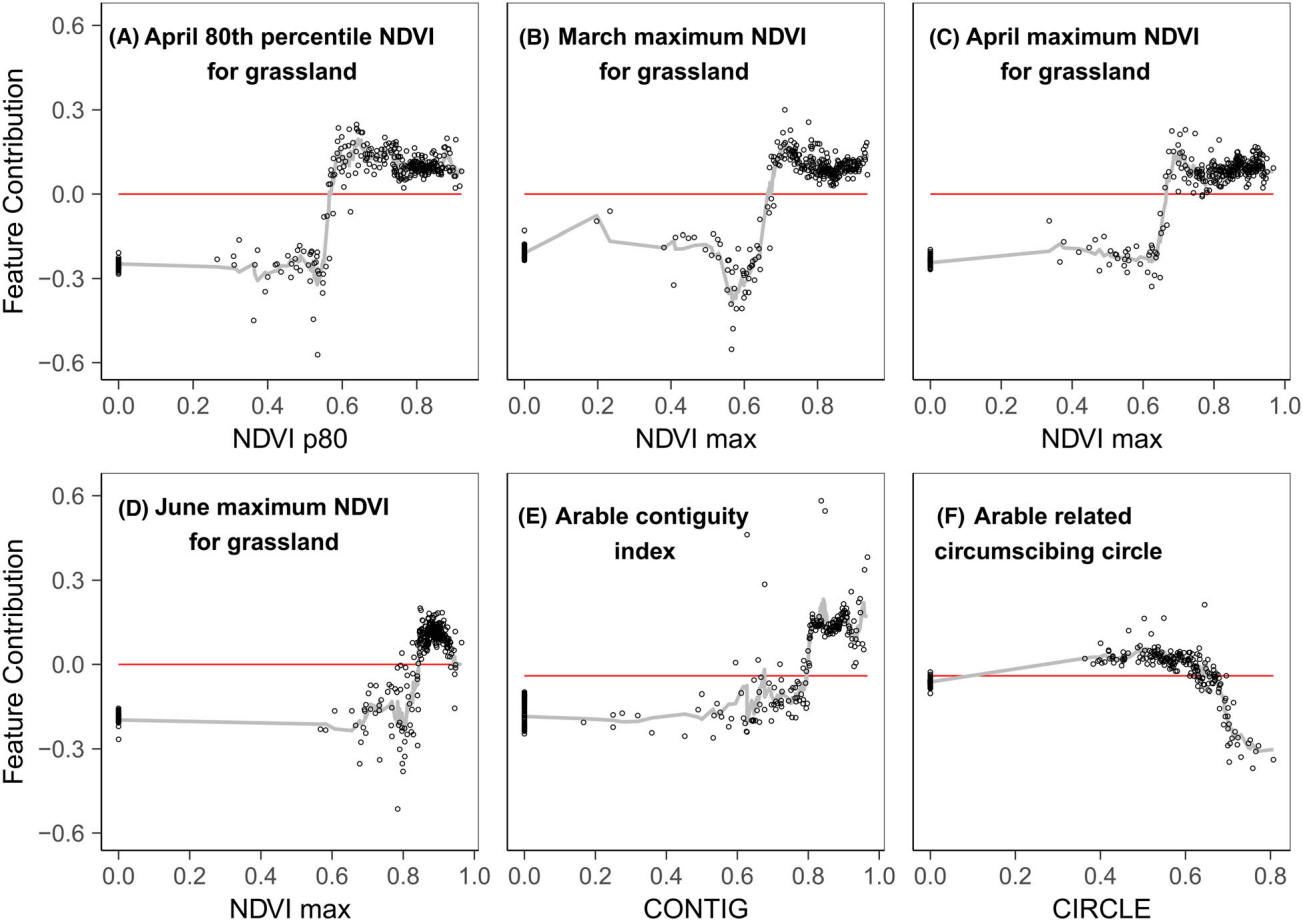
FC plots for the farmland bird diversity prediction model variables: (A) April 80th percentile NDVI for grassland, (B) March maximum NDVI for grassland, (C) April maximum NDVI for grassland, (D) June maximum NDVI for grassland, (E) arable contiguity index, and (F) arable related circumscribing circle. The *y*‐axis represents the change of predicted bird richness for a given variable value, measured with the cross‐validated FC. The *x*‐axis represents the studied variable. The fitted line is based on the *k*‐nearest neighbour (knn) estimations. The red line indicates the point of zero FC. Zero values on the *x*‐axis denote the absence of the metric from the square.

**Figure 7:**
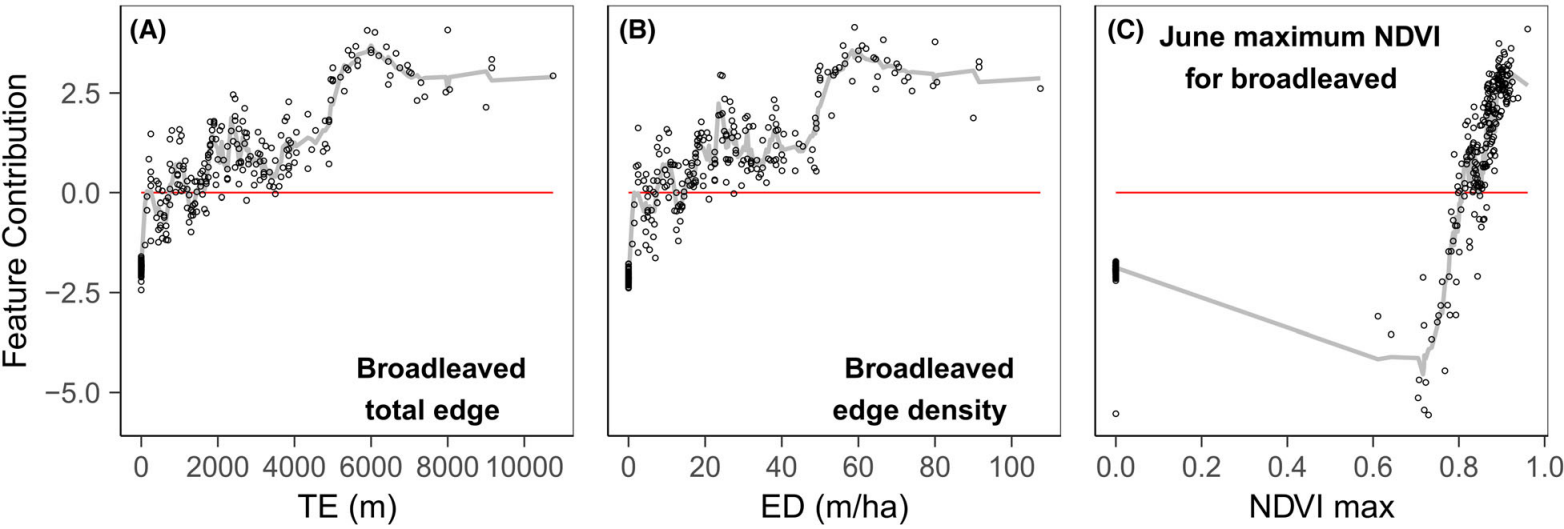
FC plots for the woodland bird richness prediction model variables: (A) broadleaved total edge, (B) broadleaved edge density, and (C) June maximum NDVI for broadleaved. The *y*‐axis represents the change of predicted bird richness for a given variable value, measured with the cross‐validated FC. The *x*‐axis represents the studied variable. The fitted line is based on the *k*‐nearest neighbour (knn) estimations. The red line indicates the point of zero FC. Zero values on the *x*‐axis denote the absence of the metric from the square.

**Figure 8:**
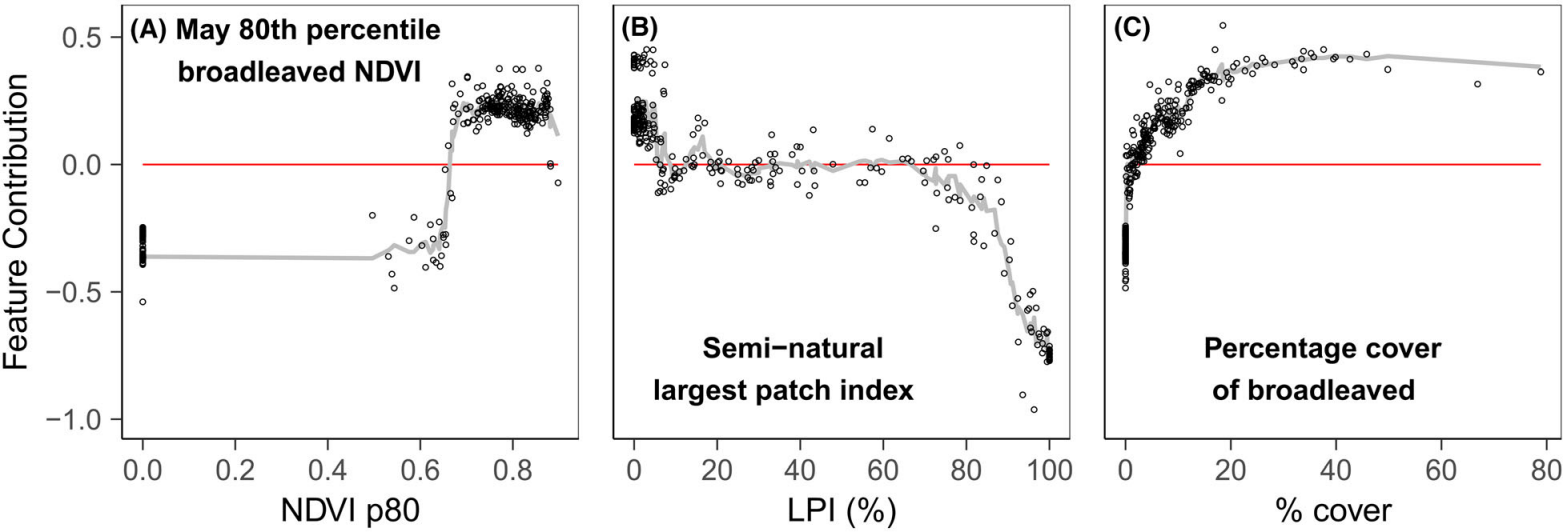
FC plots for the woodland bird diversity prediction model variables: (A) May 80th percentile broadleaved NDVI, (B) semi‐natural largest patch index, and (C) percentage cover of broadleaved woodland. The *y*‐axis represents the change of predicted bird richness for a given variable value, measured with the cross‐validated FC. The *x*‐axis represents the studied variable. The fitted line is based on the *k*‐nearest neighbour (knn) estimations. The red line indicates the point of zero FC. Zero values on the *x*‐axis denote the absence of the metric from the square.

The feature contribution analysis showed different response shapes for the different habitat productivity and heterogeneity measures, included in the refined models, for each response variable (Figs. 5, 6, 7, 8). The following section summarizes the feature contribution for each of the explanatory variables, in each refined model, for farmland bird richness and diversity, and woodland bird richness and diversity in turn. Feature contribution values above 0 indicate that a predictor value has a positive effect on the response variable; feature contribution values below 0 indicate a negative effect.

#### Farmland bird richness

The feature contribution analysis (Fig. 5) suggests that for farmland bird richness, the presence of high productivity grassland in March and April (NDVI max_Mar_ > 0.65; NDVI p80_Apr_ > 0.6) contributes positively towards richness (Fig. 5A and C), with richness peaking at NDVI values of around 0.8. Habitat fragmentation, as measured by the effective arable mesh size (see section 2.2 for details), has a positive effect on richness (Fig. 5B), with the intensity increasing rapidly at low values before plateauing from 20–30 onwards. The variation in arable NDVI in July is also important, with an NDVI range > 0.3 associated with higher bird richness.

#### Farmland bird diversity

The feature contribution analysis (Fig. 6) suggests that for farmland bird diversity, grassland productivity at key points through the year is crucial. Specifically, the presence of high productivity grassland throughout the growing season (NDVI p80_Apr_ > 0.6; NDVI max_Mar_/max_Apr_/max_Jun_ > 0.7–0.8) contributes positively towards diversity (Fig. 6). The relationships tend to increase rapidly towards a maximum before fluctuating, but generally maintain a positive influence on diversity. The pattern of arable in the landscape is also important (Figs. 6E and F), with large contiguous patches of arable land (CONTIG >0.8) promoting diversity, while very long elongated and narrow arable patches (CIRCLE >0.7) have a negative impact.

#### Woodland bird richness

The feature contribution analysis (Fig. 7) suggests that total broadleaved woodland edge length > 2000 m and edge density > 20 m/ha contribute positively towards richness, reaching peak intensity around 6000 m and 60 m/ha respectively, and flattening or reducing thereafter (Fig. 7A and B). This may indicate that the presence of more, smaller patches of broadleaved woodland or patches with more complex shapes helps to promote high richness. Broadleaved productivity in June also affects woodland richness having a negative influence below NDVI values of 0.8 and a positive, linearly increasing relationship afterwards (Fig. 7C).

#### Woodland bird diversity

For woodland bird diversity, the feature contribution analysis (Fig. 8) suggests that the presence of high productivity broadleaved woodland in May (NDVI p80_May_ > 0.7) had a positive effect on diversity, peaking around NDVI p80_May_ = 0.7 and then decreasing slightly (Fig. 8A). Additionally, it appears that having some semi‐natural land (largest semi‐natural patch index (LPI) < 10%) has a positive impact on woodland diversity, whilst very high values have negative impact, with values in‐between (LPI 10% to 80%) having little impact on diversity (Fig. 8B). The percentage cover of broadleaved woodland is also important, with cover above 5% exhibiting an increasingly positive effect on diversity, reaching a plateau at about 20%–30%.

## Discussion

### Satellite‐derived covariates

The importance of satellite imagery timing has been assessed by using individual images for different dates through the year (Bonthoux et al., 2018; Sheeren et al., 2014), whilst the temporal granularity of the satellite data has been assessed using composite images aggregated across different units of time, for example, growing season percentiles (Carroll et al., 2022) or annual values, including minimum annual NDVI (Nieto et al., 2015) or gross primary productivity (Phillips et al., 2010). There is a difference between smaller spatial scale studies (including this paper) where identifying a best time‐period may be meaningful (Bonthoux et al., 2018; Sheeren et al., 2014) and large‐scale or global studies, where it is important to produce annual globally consistent variables (Radeloff et al., 2019; Tuanmu & Jetz, 2015).

We used monthly NDVI composites, which enabled the importance of vegetation productivity at key phases of the year to be explored. For woodland birds, the NDVI for broadleaved woodland was important, with May and June being the key months. This is similar to Hawkins (2004) who found summer vegetation‐indexes better for predicting bird richness than annual values, whereas other studies have found autumn best for woodland bird species richness (Bonthoux et al., 2018; Sheeren et al., 2014). However, neither of these studies used habitat specific NDVI values; instead, they used coarser resolution, pixel data (250 m or 1 km), which will primarily reflect the dominant habitat (Bonthoux et al., 2018).

For farmland birds, we found that April and March grassland NDVI were the most important covariates. Yamaura et al. (2010) also found that spring greenness was important for bird species mapping in Japan, whereas Bonthoux et al. (2018) found that satellite data from early March produced the least accurate predictions, with images from the end of June best for farmland birds. Previous work has shown strong correlations between the NDVI for spring green‐up of grassland and annual grassland productivity (Tebbs et al., 2017) and with arthropod abundance (Fernández‐Tizón et al., 2020) suggesting links to energy/food resources (Bonn et al., 2004). The number of studies looking into monthly/seasonal satellite data for bird mapping is still relatively limited, and many of the existing studies are coarse scale (250 m or larger pixel size). Further work is required to assess the stability of our results to see whether the types of metrics, habitats and months that were important here remain consistent between years.

The spatial granularity used in biodiversity studies is often the individual pixel, with spatial context provided by image texture (St‐Louis et al., 2006) or landscape structure metrics (Atauri & de Lucio, 2001). Information on landscape structure (including composition and configuration) is included in bird mapping to improve estimates of biodiversity patterns and to test ecological hypotheses, such as understanding the relative roles of habitat configuration and extent (Basile et al., 2021; Suárez‐Castro et al., 2022). Here, we used 1 km squares as our modelling resolution, with higher resolution data to quantify landscape composition and configuration, along with habitat‐specific NDVI to provide information on habitat quality. The use of higher resolution data is important as previous work has shown that higher spatial resolution data tends to produce better estimates of bird biodiversity (Hurlbert & Haskell, 2003; Sheeren et al., 2014), possibly by enabling more precise estimates of the environmental conditions that affect birds. Although key scales may vary with species and habitat, as the processes that regulate species richness are scale dependent (Hurlbert & Haskell, 2003; Luoto et al., 2007).

To quantify within habitat productivity and heterogeneity, we used NDVI metrics, that included a subset of the first order image texture metrics (e.g. range, standard deviation), which many studies have found important for biodiversity mapping (Farwell et al., 2021; St‐Louis et al., 2006; Tuanmu & Jetz, 2015). The spatial extent of texture metrics is typically defined by a filter window around a central pixel (see for example St‐Louis et al., 2006 who test windows from 3 × 3 to 101 × 101 pixels). Image texture calculated for a filter window covering a single habitat captures within class heterogeneity (Culbert et al., 2012; Farwell et al., 2021); however, metrics calculated over a mixed pixel, or mixed filter window, will mainly reflect the dominant habitat type (Bonthoux et al., 2018). In areas with more fragmented land cover like the UK (Hill & Smith, 2005), land cover specific metrics may be useful to prevent mixing signals from different land cover types. So, here we used habitat‐specific monthly NDVI values to capture within habitat heterogeneity and productivity. In our results habitat‐specific productivity was the most important variable in all the final models, except for woodland bird richness. Habitat‐specific NDVI values are not often calculated for biodiversity mapping; however, our results show they are important in some landscapes and so should be explored more widely, including in comparison with the more widely used image texture methods. Habitat heterogeneity is strongly influenced by scale (Rocchini, 2007), so the relative importance of habitat‐specific metrics versus image texture is likely to be context dependent.

Remote sensing for biodiversity mapping is an active, well‐established area of research. However, considerable development is still required to produce operational integrated biodiversity monitoring systems covering comprehensive spatial and taxonomic extents (Jetz et al., 2019). Part of this development is improved understanding of the covariates that are important for different species. However, in the literature, it is difficult to separate general results from specific results, especially when comparing studies with different sets of covariates, across different species, ranges of species and different spatiotemporal extents. This is compounded by the spatial and temporal variability in the quality of satellite‐derived covariates. Therefore, to consolidate work in this area it may be timely for a comprehensive review of environmental covariates for avian biodiversity to identify knowledge gaps building on earlier work (Gottschalk et al., 2005), broader reviews (e.g. Jetz et al., 2019; Wüest et al., 2019) and initiatives like the development of biodiversity‐focused indices (Radeloff et al., 2019) and the essential biodiversity variables (Jetz et al., 2019).

### Influence of landscape heterogeneity and productivity on bird diversity

There is ongoing debate about the relative roles of habitat quantity versus habitat structure (fragmentation/connectivity) for biodiversity (Fletcher et al., 2018). In this study we calculated habitat heterogeneity (FRAGSTATS), including metrics of habitat extent and habitat configuration for 1 km squares. Habitat extent was only important in the woodland bird diversity model, but measures of habitat configuration were important in all the final models, suggesting that, at least in this case, habitat arrangement is more important. Like previous work (Tu et al., 2020), the habitat extent and configuration variables used in the final richness and diversity models are different (Table 3). From a conservation perspective, this suggests that different interventions might be required depending on whether the aim is to increase bird diversity or bird richness. Here, we used aggregated groups of birds, however, to produce evidence on specific spatial drivers for conservation interventions, modelling individual species might provide more insight.

The woodland bird richness model is the only one where habitat heterogeneity variables are more important than the productivity variables. The feature contribution analysis shows that woodland bird richness was higher in areas with longer woodland edges, supporting previous studies showing bird richness and diversity is greatly enhanced by open habitats within woodlands and woodland edges (Fuller, Smith, et al., 2007; Terraube et al., 2016). Higher plant diversity and composition, and habitat structural diversity at woodland edges, have been proposed as possible drivers of bird diversity within these habitats (Carrasco et al., 2019; Terraube et al., 2016).

The feature contribution indicates that farmland bird richness is higher in areas where arable land is less fragmented (i.e. higher values of effective mesh size, although this positive effect stabilizes at an effective mesh size of around 30). However, for both the farmland richness and diversity models, the first variable is grassland productivity‐based illustrating the importance of grassland habitats in supporting GB farmland birds. This reflects earlier work on the habitat preferences of bird species, which found that links between farmland birds and arable habitats alone were relatively weak (Fuller et al., 2005). In part, because farmland birds showed associations with arable areas, but also with other lowland habitats (Fuller, Devereux, et al., 2007), providing additional ecological niches (Fuller et al., 2004). However, such effects are likely to be scale‐dependent and influenced by landscape structure beyond the 1 km square (Robinson et al., 2001).

### Broader implications for conservation

Conservationists have a range of tools that can be deployed to protect species, however, they need to be supported by accurate evidence. In the UK, this evidence‐base has been provided by the Breeding Bird Survey data, which shows the spatial and temporal changes in bird populations. From a conservation perspective, it is important to be able to identify where birds are stable and where they are declining, which then informs decisions about when and where to intervene. The methods here provide a useful tool for understanding how different spatial drivers influence bird distributions. However, to be of operational use for conservation science, the method needs to be applied slightly differently and developed further, specifically through the following:

#### Assessing stability over time

In the example here, the method was applied to data for 2000. However, it is likely that the quality of the satellite data and the bird data will vary from year to year. So it is important to assess the stability of the results over time and quantify the uncertainty in the models to give a more robust understanding of both the key spatial drivers and their relationship with bird richness and diversity. Whilst some stability over shorter time‐scales will confer credibility on the model outputs, on longer time‐scales the spatial drivers would be expected to change, as bird species respond to climate change and to large‐scale landscape changes like afforestation.

#### Increasing model interpretability

Recent developments in machine learning have stressed the importance of increasing the interpretability of machine learning models, including their inputs (Zytek et al., 2022). Machine learning models typically use data collected by sensors or satellites; however, this data is not always readily understandable by users in the field. In our case, one model shows that June maximum broadleaved NDVI is important, however, it would be more beneficial to conservationists if we used known sites to develop a taxonomy (or dictionary) that translated this into a set of likely traits for such woodland, for example in terms of likely tree maturity, tree canopy cover and possibly species mix. The same is true for the grassland variables, for instance, intense management of grasslands can reduce the suitability of grassland habitats to provide breeding and foraging resources for birds (Vickery et al., 2001), but evidence is needed to link such management to the NDVI metrics seen in the models. This is also likely to highlight the similarity in some of the variables.

Machine learning and big data techniques have a role to play in biodiversity and conservation science (Wüest et al., 2019). In this study, we used feature importance to identify the most important features in the four final models and then feature contribution to provide insight into the relationship between individual covariates and bird richness and diversity. The feature contribution enables the identification of thresholds and ranges in key variables that strongly positively (or negatively) affect bird richness and diversity. For example, the June maximum NDVI feature contribution result shows that whilst NDVI >0.8 has a positive effect on farmland bird diversity, this peaks at NDVI ~0.9, but then drops for higher values. This potentially provides more insight into the features, and crucially the level of a particular feature, that positively affect bird richness and diversity than has typically been provided by machine learning based methods (e.g. the threshold‐based outputs from individual decision trees (Coops, Wulder, et al., 2009)). Feature contribution provides a useful tool for exploring the variables important to modelling bird richness or diversity, but it will be crucial to assess the stability of these relationships over time, and for other regions, to understand to what extent they can inform conservation activities.

#### Developing data and tools for conservation

Advances such as Google Earth Engine (Gorelick et al., 2017) make it timely to reconsider how best to facilitate wider use of satellite data in bird distribution modelling. For example, this might be through the production and curation of key satellite metrics, like monthly NDVI, or easy to use tools to derive them. The metrics could be routinely created (in the same way that climate variables are) for conservationists and ecologists to use in their models. Additionally, whilst the maps here have been validated at GB‐level, it would be beneficial to conduct a landscape‐level validation, to quantify how well the maps detect spatial patterns in bird distributions at a fine‐scale.

## Conclusion

This study shows that habitat‐specific monthly NDVI values, alongside landscape structure (FRAGSTATS) metrics, are useful for predicting farmland and woodland bird diversity and richness (*R* ^2^ 0.64 to 0.77) in GB. The variable selection highlights a number of important spatial drivers of bird species richness and diversity including high productivity grassland during spring for farmland birds and woodland patch edge length for woodland birds. Meanwhile, feature contribution provides information on the form (magnitude and direction) of the relationships between these environmental variables and bird richness and diversity. This work demonstrates that machine learning tools, like feature contribution, have the potential to provide insights into the spatial drivers of wildlife community structure.

## Author Contributions

MH, AB and CR conceived the ideas and designed the methodology; GS provided access to the bird data; MH analysed the data, with support from LC; MH, AB and CR interpreted the results and led the writing of the manuscript; all authors revised the draft manuscript and approved the final version.

## Data Accessibility Statement

LCM2000 data are deposited at https://doi.org/10.5285/d5ee5360-12c5-448c-9d2b-f5c941fe3948 (Fuller et al., 2002). Countryside Survey data are accessible via https://countrysidesurvey.org.uk/. Due to confidentiality of location data, spatial information is available subject to a licence agreement. Details are available here: https://countrysidesurvey.org.uk/data. Landsat data are available for free via USGS Earth Explorer (https://earthexplorer.usgs.gov/). In this study the data were accessed through Google Earth Engine (https://earthengine.google.com/) using the LANDSAT/LE07/C01/T1_SR and LANDSAT/LT05/C01/T1_SR collections.

## Supporting information


**Table S1.** Broad land cover classes used in this study and the corresponding original LCM2015 subclasses.
**Table S2.** Details of the habitat structure metrics derived from LCM2015 using FRAGSTATS. Metric descriptions based on McGarigal (2015).
**Table S3.** Variables categorised as being important by minimal depth selection for each response variable.
**Table S4.** Correlation coefficients for predictor variables in the final refined models for (i) farmland bird richness, (ii) farmland bird diversity, (iii) woodland bird richness, and (iv) woodland bird diversity.
