# Supplementary material for: Using satellite data to assess spatial drivers of bird diversity
Source: Remote Sens Ecol Conserv. 2022 Dec 24;9(4):483–500. doi: 10.1002/rse2.322 (PMC10946777; doi:10.1002/rse2.322)
Supplement: Supplementary file 1 — Table S1. Broad land cover classes used in this study and the corresponding original LCM2015 subclasses. Table S2. Details of the habitat structure metrics derived from LCM2015 using FRAGSTATS. Metric descriptions based on McGarigal (2015). Table S3. Variables categorised as being important by minimal depth selection for each response variable. Table S4. Correlation coefficients for predictor variables in the final refined models for (i) farmland bird richness, (ii) farmland bird diversity, (iii) woodland bird richness, and (iv) woodland bird diversity. [file RSE2-9-483-s001.docx]

**Table S1: Broad land cover classes used in this study and the corresponding original LCM2015 subclasses.**

| **LC class** | **LCM Subclasses** |
| --- | --- |
| Arable | Arable cereals, arable horticulture, non-rotational horticulture |
| Broad-leaved | Broad-leaved/mixed woodland |
| Coniferous | Coniferous woodland |
| Grassland | Improved grassland, set-aside grassland, neutral grassland, calcareous |
| Semi-natural | Acid grassland, bracken, dense and open dwarf shrub heath, fen, marsh, swamp, bogs (deep peat), montane habitats, inland bare ground, saltmarsh |
| Urban | Continuous urban, suburban/rural developed |
| Water | Water (inland) |
| Coast | Supra-littoral rock, supra-littoral sediment, littoral rock, littoral sediment |
| Sea | Sea/estuary |

**Table S2: Details of the habitat structure metrics derived from LCM2015 using FRAGSTATS. Metric descriptions based on McGarigal (2015).**

| **Variable** | **Abbreviation (Unit)** | **Description** |
| --- | --- | --- |
| ***Area & Edge Metrics*** | | |
| Area | AREA_AM/CV/MN_ (ha) | Area of each patch comprising a landscape mosaic given as mean (MN), coefficient of variation (CV) or area-weighted mean (AM) per class. |
| Percentage of landscape | PLAND (%) | Percentage of the landscape comprised of a particular patch type |
| Edge Density | ED (m/ha) | Edge length of a particular patch type standardised to a per unit area basis. |
| Radius of Gyration | GYRATE_AM/CV/MN_ (m) | Measure of patch extent given as mean (MN), coefficient of variation (CV) or area-weighted mean (AM) per class. |
| Largest Patch Index | LPI (%) | Quantifies the percentage of the total landscape area comprised by the largest patch. |
| Total Edge | TE (m) | Absolute measure of total edge length of a particular patch type. |
| ***Shape Metrics*** | | |
| Related Circumscribing Circle | CIRCLE_AM/CV/MN_ | Measure of overall patch elongation using the ratio of patch area to the ratio of the smallest circumscribing circle given as mean (MN), coefficient of variation (CV) or area-weighted mean (AM) per class. |
| Contiguity Index | CONTIG_AM/CV/MN_ | Measure of spatial connectedness/contiguity of cells within a grid-cell given as the mean (MN), coefficient of variation (CV) or area-weighted mean (AM) per class. |
| Fractal Dimension Index | FRAC_AM/CV/MN_ | Measure of shape complexity given as a mean (MN), coefficient of variation (CV) or area-weighted mean (AM) per class. |
| Perimeter-Area Ratio | PARA_AM/CV/MN_ | Ratio of patch perimeter to area given as mean (MN), coefficient of variation (CV) or area-weighted mean (AM) per class, providing a measure of shape complexity. |
| Shape Index | SHAPE_AM/CV/MN_ | Measures the complexity of patch shape compared to a standard shape (square) of the same size. Values are given as mean (MN), coefficient of variation (CV) or area-weighted mean (AM) per class. |
| ***Aggregation Metrics*** | | |
| Patch Cohesion | COHESION | Provides a measure of the physical connectedness of the corresponding patch types. |
| Landscape Division Index | DIVISION (proportion) | Probability that 2 randomly chosen pixels in the landscape are not situated in the same undissected patch of the corresponding patch type. |
| Effective Mesh Size | MESH (ha) | Quantifies habitat fragmentation based on the probability that two randomly chosen points in the region under interest are located in the same non-fragmented patch (Jaeger, 2000). The probability is multiplied by the total area of the landscape unit. |
| Number of Patches | NP | Number of patches of a particular patch type |
| Patch Density | PD (number of patches per 100 ha) | Number of patches of the corresponding patch type standardised on a per unit area basis |
| Splitting Index | SPLIT | SPLIT is 1 when the landscape consists of a single patch, increasing in value as the focal patch type is increasingly reduced in area and subdivided into smaller patches. |

**Table S3: Variables categorised as being important by minimal depth selection for each response variable.**

| **Response Variable** | **Predictor Variables** | | |  |
| --- | --- | --- | --- | --- |
| Farmland Bird Richness | Percentage cover of arable land  April maximum NDVI for semi-natural  Grassland perimeter-area ratio  June median NDVI for grassland  June mean NDVI for grassland  April maximum NDVI for grassland  Arable edge density | August minimum NDVI for grassland  September maximum NDVI for able  Broadleaved related circumscribing circle  September NDVI coefficient of variation for arable  August NDVI range for grassland | | NDVI: 14  FRAGSTATS: 6 |
| Farmland Bird Diversity | April 80^th^ percentile NDVI for grassland  March maximum NDVI for grassland  April maximum NDVI for grassland  June maximum NDVI for grassland  Arable contiguity index  Arable related circumscribing circle  May maximum NDVI for grassland  June 80^th^ percentile NDVI for grassland | | March 80^th^ percentile NDVI for grassland  May 80^th^ percentile NDVI for grassland  Percentage cover of urban  April median NDVI for grassland  Percentage cover of coast  April 20^th^ percentile NDVI for grassland  April mean NDVI for grassland  March median NDVI for grassland | NDVI: 12  FRAGSTATS: 4 |
| Woodland Bird Richness | Broadleaved total edge length  Broadleaved edge density  June maximum NDVI for broadleaved  Percentage cover of broadleaved  May 80^th^ percentile NDVI for broadleaved  Semi-natural largest patch index  May maximum NDVI for broadleaved  June 80^th^ percentile NDVI for broadleaved  March maximum NDVI for grassland | | Semi-natural patch cohesion  March 80^th^ percentile NDVI for grassland  September maximum NDVI for broadleaved  Semi-natural effective mesh size  May median NDVI for broadleaved  September NDVI inter-quartile range for semi-natural  April maximum NDVI for grassland  July NDVI range for broadleaved  September 80^th^ percentile NDVI for semi-natural | NDVI: 12  FRAGSTATS: 6 |
| Woodland Bird Diversity | May 80^th^ percentile NDVI for broadleaved  Semi-natural largest patch index  Percentage cover of broadleaved  June 80^th^ percentile NDVI for broadleaved  June maximum NDVI for broadleaved  Semi-natural patch cohesion  Broadleaved total edge length  Semi-natural effective mesh size  May median NDVI for broadleaved  March maximum NDVI for grassland  May maximum NDVI for broadleaved  July maximum NDVI for semi-natural  Broadleaved edge density  Percentage cover of semi-natural  July NDVI range for broadleaved  March 80^th^ percentile NDVI for grassland  September NDVI inter-quartile range for semi-natural  August NDVI standard deviation for semi-natural  Broadleaved radius of gyration | | May 80^th^ percentile NDVI for coniferous  Semi-natural contiguity index  June median NDVI for broadleaved  August 20^th^ percentile NDVI for semi-natural  Broadleaved effective mesh size  Semi-natural average patch size  June mean NDVI for broadleaved  September 80^th^ percentile NDVI for semi-natural  July NDVI standard deviation for broadleaved  May median NDVI for coniferous  July 80^th^ percentile NDVI for broadleaved  September 80^th^ percentile NDVI for broadleaved  Broadleaved largest patch index  August mean NDVI for semi-natural  Broadleaved average patch size  Grassland contiguity index  April NDVI inter-quartile range for coniferous  September maximum NDVI for broadleaved | NDVI: 23  FRAGSTATS: 14 |

**Table S4: Correlation coefficients for predictor variables in the final refined models for (i) farmland bird richness, (ii) farmland bird diversity, (iii) woodland bird richness, and (iv) woodland bird diversity.**

| **(i) Farmland bird richness** | **March maximum grassland NDVI** | **Effective arable mesh size** | **April 80th percentile grassland NDVI** | **July range arable NDVI** |
| --- | --- | --- | --- | --- |
| **March maximum grassland NDVI** | 1 | 0.18 | 0.97 | 0.57 |
| **Effective arable mesh size** | 0.18 | 1 | 0.2 | 0.49 |
| **April 80th percentile grassland NDVI** | 0.97 | 0.2 | 1 | 0.58 |
| **July range arable NDVI** | 0.57 | 0.49 | 0.58 | 1 |

| **(ii) Farmland bird diversity** | **April 80th percentile grassland NDVI** | **March maximum grassland NDVI** | **April maximum grassland NDVI** | **June maximum grassland NDVI** | **Arable contiguity index** | **Arable related circumscribing circle** |
| --- | --- | --- | --- | --- | --- | --- |
| **April 80th percentile grassland NDVI** | 1 | 0.97 | 0.99 | 0.94 | 0.65 | 0.64 |
| **March maximum grassland NDVI** | 0.97 | 1 | 0.98 | 0.96 | 0.63 | 0.63 |
| **April maximum grassland NDVI** | 0.99 | 0.98 | 1 | 0.96 | 0.62 | 0.63 |
| **June maximum grassland NDVI** | 0.94 | 0.96 | 0.96 | 1 | 0.56 | 0.57 |
| **Arable contiguity index** | 0.65 | 0.63 | 0.62 | 0.56 | 1 | 0.92 |
| **Arable related circumscribing circle** | 0.64 | 0.63 | 0.63 | 0.57 | 0.92 | 1 |

| **(iii) Woodland bird richness** | **Broadleaved total edge** | **Broadleaved edge density** | **June maximum broadleaved NDVI** |
| --- | --- | --- | --- |
| **Broadleaved total edge** | 1 | 1 | 0.65 |
| **Broadleaved edge density** | 1 | 1 | 0.65 |
| **June maximum broadleaved NDVI** | 0.65 | 0.65 | 1 |

| **(iv) Woodland bird diversity** | **May 80th percentile broadleaved NDVI** | **Semi-natural largest patch index** | **Percentage broadleaved woodland** |
| --- | --- | --- | --- |
| **May 80th percentile broadleaved NDVI** | 1 | -0.61 | 0.45 |
| **Semi-natural largest patch index** | -0.61 | 1 | -0.32 |
| **Percentage broadleaved woodland** | 0.45 | -0.32 | 1 |
